# A *fructan*: the *fructan 1-fructosyl-transferase* gene from *Helianthus tuberosus* increased the PEG-simulated drought stress tolerance of tobacco

**DOI:** 10.1186/s41065-020-00131-3

**Published:** 2020-04-20

**Authors:** Xuemei Sun, Yuan Zong, Shipeng Yang, Lihui Wang, Jieming Gao, Ying Wang, Baolong Liu, Huaigang Zhang

**Affiliations:** 1 Key Laboratory of Adaptation and Evolution of Plateau Biota, Northwest Institute of Plateau Biology, The Innovative Academy of Seed Design, Chinese Academy of Sciences, Xining, 810001 China; 2 Qinghai Province Key Laboratory of Crop Molecular Breeding, Xining, 810001 China; 3 University of Chinese Academy of Sciences, Beijing, 100049 China; 4 Qinghai Province Key Laboratory of Vegetable Genetics and Physiology, Xining, 810016 China; 5 Academy of Agriculture and Forestry Sciences, Qinghai University, Xining, 810016 China

**Keywords:** *Helianthus tuberosus*, *Fructan*, *fructan 1-fructosyltransferase* (*1-FFT*), PEG-simulated drought stress

## Abstract

**Background:**

Jerusalem artichoke (*Helianthus tuberosus*) is a fructan-accumulating plant, and an industrial source of raw material for fructan production, but the crucial enzymes involved in fructan biosynthesis remain poorly understood in this plant.

**Results:**

In this study, a *fructan: fructan 1-fructosyl-transferase* (*1-FFT*) gene, *Ht1-FFT*, was isolated from Jerusalem artichoke. The coding sequence of Ht1-FFT was 2025 bp in length, encoding 641 amino acids. Ht1-FFT had the type domain of the 1-FFT protein family, to which it belonged, according to phylogenetic tree analysis, which implied that Ht1-FFT had the function of catalyzing the formation and extension of beta-(2,1)-linked fructans. Overexpression of *Ht1-FFT* in the leaves of transgenic tobacco increased fructan concentration. Moreover, the soluble sugar and proline concentrations increased, and the malondialdehyde (MDA) concentration was reduced in the transgenic lines. The changes in these parameters were associated with increased stress tolerance exhibited by the transgenic tobacco plants. A PEG-simulated drought stress experiment confirmed that the transgenic lines exhibited increased PEG-simulated drought stress tolerance.

**Conclusions:**

The *1-FFT* gene from *Helianthus tuberosus* was a functional *fructan: fructan 1-fructosyl-transferase* and played a positive role in PEG-simulated drought stress tolerance. This transgene could be used to increase fructan concentration and PEG-simulated drought stress tolerance in plants by genetic transformation.

## Background

Fructans are the third most-common storage carbohydrate in higher plants, currently found in about 15% of flowering plants [1, 2]. In addition to being an important storage carbohydrate for plants, fructans are also important for plants to tolerate environmental stresses [3, 4]. Fructan-accumulating species predominate in cold and dry environments, but hardly exist in tropical or aquatic environments [5]. The drought tolerance traits of *Echinacea rigida* and *Chrysanthemum verum* are extremely high, which are related to their accumulation of fructans with a high degree of polymerization. Fructan concentration in the roots and leaves of chicory (*Cichorium intybus*) seedlings exposed to drought was significantly higher than that of control seedlings, indicating that fructan metabolism was involved in drought tolerance [6]. Fructans participate in plant abiotic stress tolerance mainly through osmotic regulation and the maintenance of membrane stability [7]. The hexose produced by fructan hydrolysis can reduce the freezing point of liquid water in cells and allow the sustained growth of leaves under drought conditions, as well as reducing the phase transition temperature of membranes and stabilizing the phospholipid bilayer [8–11].

The pathway of fructan biosynthesis is relatively well understood. Four fructosyltransferases (FTs) are considered to be involved in fructan biosynthesis [12]. They are sucrose: sucrose-1- fructosyl transferase (1-SST) [13, 14], sucrose: fructose-6-fructosyltransferase (6-SFT) [15], fructose: fructose-1-fructosyltransferase (1-FFT) [16], and fructose: fructose-6G-fructosyltransferase (6G-FFT) [17]. Depending on the donor matrix, the FTs can be divided into two types: S-type FTs (1-SST and 6-SFT, with sucrose as the donor matrix) and F-type FTs (1-FFT and 6G-FFT, with fructose as the donor matrix) [18]. FTs are generally believed to originate from sucrose invertase. The main structures of these proteins are highly similar, and these proteins belong to the plant glycoside hydrolase family GH32 [19]. Overexpression of FTs can improve the stress tolerance of fructan-non-accumulating crops, such as tobacco [20], beet [21, 22] and potato [23].

Jerusalem artichoke (*Helianthus tuberosus* L.) is a typical fructan-accumulating plant, which makes it an important industrial raw material for fructan production [24]. Because of its considerable stress tolerance, Jerusalem artichoke is mainly planted in arid and coastal saline-alkali areas of Northwest China. The FTs from Jerusalem artichoke should have high catalytic activity for fructan biosynthesis [25]. In the current study, *Ht1-FFT* was isolated from Jerusalem artichoke, and its molecular characteristics were analyzed. Through transforming tobacco with *Ht1-FFT*, it was found that the PEG-simulated drought tolerance of the transgenic plants was increased. The results should be helpful in verifying the function of *Ht1-FFT*, and to understanding the mechanism by which fructose metabolism increases abiotic stress tolerance.

## Results

### Isolation of *Ht1-FFT* from Jerusalem artichoke

**Fig. 1:**
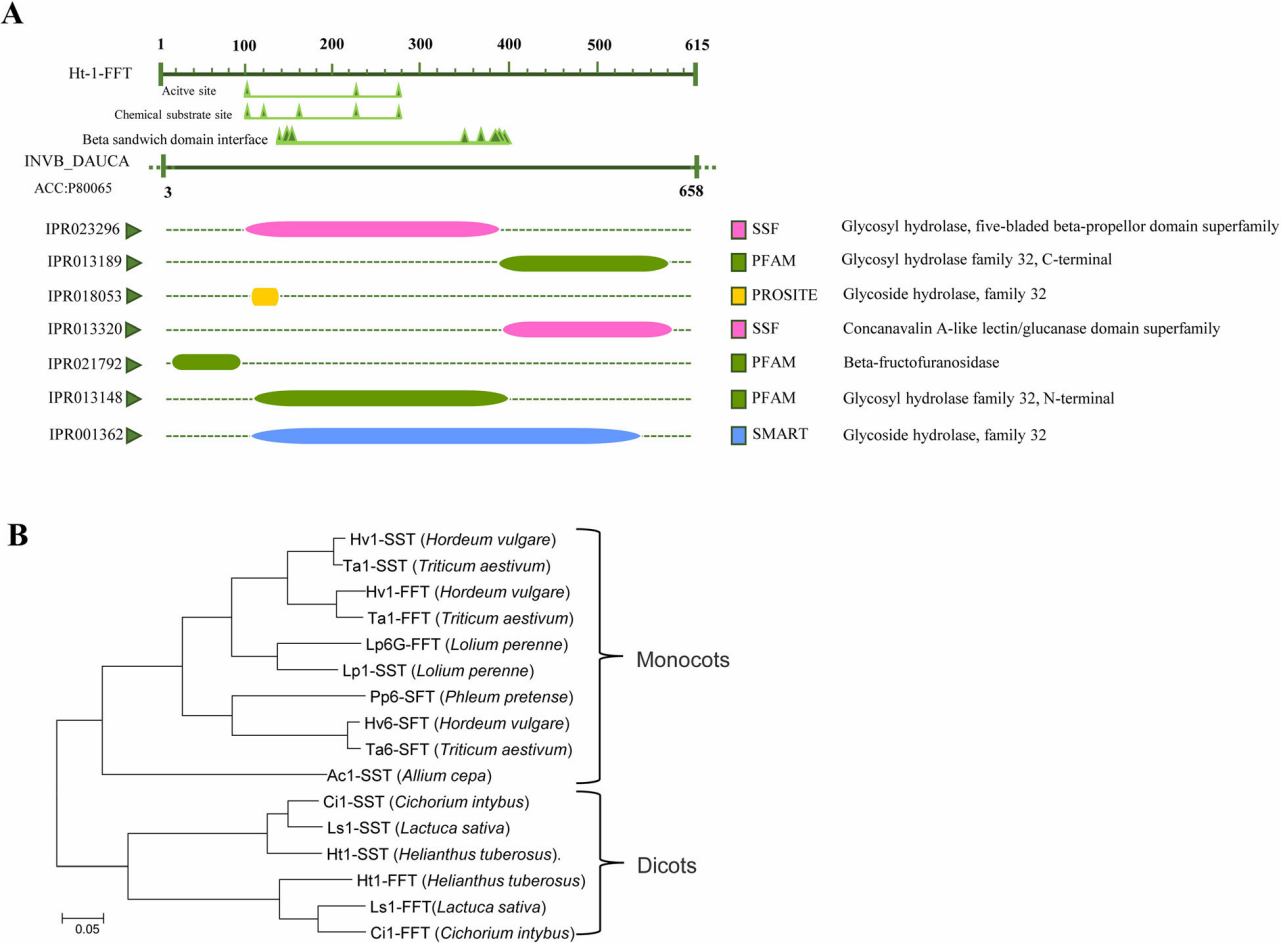
The molecular characteristics of *Ht1-FFT* from Jerusalem artichoke. **a** The conserved domain prediction of Ht1-FFT. **b** The phylogenetic tree of FTs involved in fructan biosynthesis. The log-in numbers of these proteins (or translated products) in the GenBank database are as follows: *Helianthus tuberosus* Ht6-SFT: CAA08812; *Helianthus tuberosus* Ht1-FFT: CAA08811; *Allium cepa* Ac1-SST: BAU46555; *Phleum pratense* Pp6-SFT: BAH30252; *Lolium perenne* Lp1-SST: BAF99807; *Triticum aestivum* Ta1-FFT: BAE19751; *Triticum aestivum* Ta6-SFT: BAB82469; *Triticum aestivum* Ta1-SST: AMZ79593; *Hordeum vulgare* Hv6-SFT: AFP72240; *Hordeum vulgare* Hv1-FFT: AFP72239; *Hordeum vulgare* Hv1-SST: AFP72238; *Cichorium intybus* Ci1-FFT: AFB83199; *Lolium perenne* Lp6G-FFT: AF492836_1; *Lactuca sativa* Ls1-FFT: ABX90020; *Lactuca sativa* Ls1-SST: ABX90019; *Cichorium intybus* Ci1-SST: AAB58909

The full-length CDS of *Ht1-FFT* was 2025 bp and encoded 641 amino acids (Fig. S1). Ht1-FFT contained the intact active site, the substrate-binding site and the beta-sandwich domain interface, which was important for the fructosidase domain-containing protein to carry out its catalytic activity (Fig. 1a). Further protein annotation showed that Ht1-FFT carried the domain of beta-fructofuranosidase, and the N-terminal, and C-terminal sequences of glycoside hydrolase family 32 in the Pfam database. According to the SSF database, the five-bladed beta-propeller domain of glycoside hydrolases, and the concanavalin A-like lectin/glucanase domain were found in Ht1-FFT. It was also shown to be homologous to the glycoside hydrolase family 32 in the PROSITE database. These results indicate that Ht1-FFT belongs to glycoside hydrolase family 32 (Fig. 1a). As is already known, *Hordeum vulgare*, *Triticum aestivum*, *Lolium perenne*, *Phleum pratense*, *Allium cepa*, *Cichorium intybus*, *Lactuca sativa*, and *Helianthus tuberosus* are examples of fructan-accumulating plant species. FTs were selected from these species to construct the phylogenetic tree. Ht1-FFT was closest to the 1-FFTs from *L. sativa* and *C. intybus* (Fig. 1b). Interestingly, the 1-FFTs and 1-SSTs of the dicots occupied the same branch of the tree, whereas the FTs of monocots belonged to the other branch (Fig. 1b). The taxonomic relationship among the species played an important role in the phylogenetic relationship of 1-FFT and 1-SST, which meant that *1-FFT* and *1-SST* diverged after the divergence of dicots and monocots.

### The phenotype of transgenic tobacco exposed to PEG-simulated drought stress

**Fig. 2:**
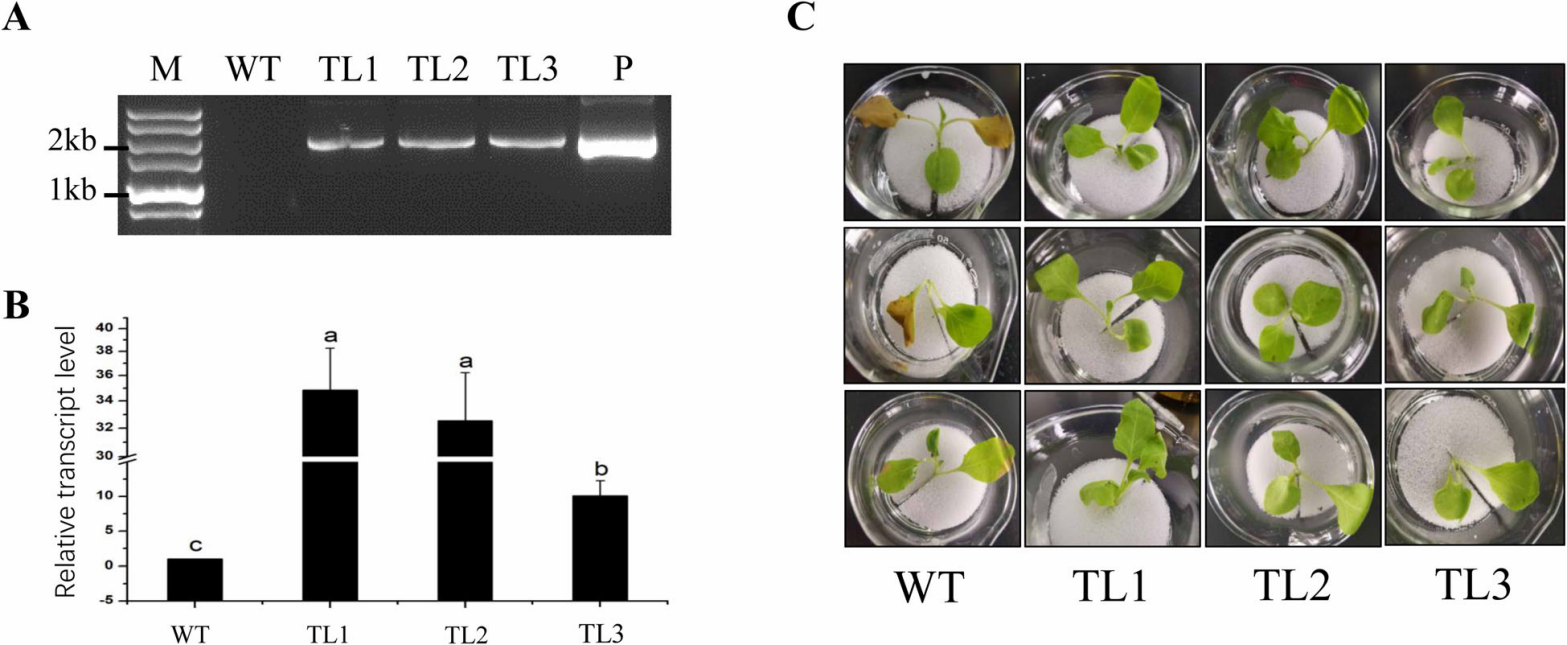
The identification of transgenic tobacco lines and their phenotype under PEG-simulated drought stress. **a** The electrophoresis of the product after PCR amplification of the *Ht1-FFT* gene in transgenic and WT tobacco lines. Lanes: M: DNA marker; WT: wild type; TL1 to TL3: transgenic lines; P: positive control (plasmid). **b** The mean ± SE relative transcript level of *Ht1-FFT* in the leaves of transgenic lines, using actin gene as the internal control. WT: wild type; TL1 to TL3: transgenic lines; any two lines with a different lowercase letter are significantly different (*P* < 0.05), using Tukey’s honestly significant difference. **c** Tobacco seedlings under PEG-simulated drought stress treatment (20% PEG). WT: wild type; TL1 to TL3: putative transgenic lines

The presence of *Ht1-FFT* in the transgenic tobacco plants was evaluated by PCR and semiquantitative RT-PCR analysis. The expected band was amplified from the positive transformants, whereas no band was obtained from the wild-type (WT) tobacco (Fig. 2a). RT-PCR experiments showed that the expression levels of *Ht1-FFT* in the transgenic tobacco were significantly higher than that in WT plants (Fig. 2b). The PEG-simulated drought stress tolerance of the transgenic tobacco was compared with that of WT tobacco. After exposure to PEG-simulated drought stress conditions for 16 h, under 10% or 15% PEG stress, WT plants wilted slightly, whereas transgenic tobacco plants did not. However, under 20% PEG stress, the leaves of WT tobacco plants turned yellow and wilted, while the leaves of transgenic tobacco remained green, with normal turgidity status (Fig. 2c). The physiological parameters of plants in response to treatment with 20% PEG stress were measured in subsequent experiments to evaluate the PEG-simulated drought tolerance of transgenic tobacco expressing *Ht1-FFT*.

### The physiological parameters of transgenic *Ht1-FFT* tobacco in response to 20% PEG stress

**Fig. 3:**
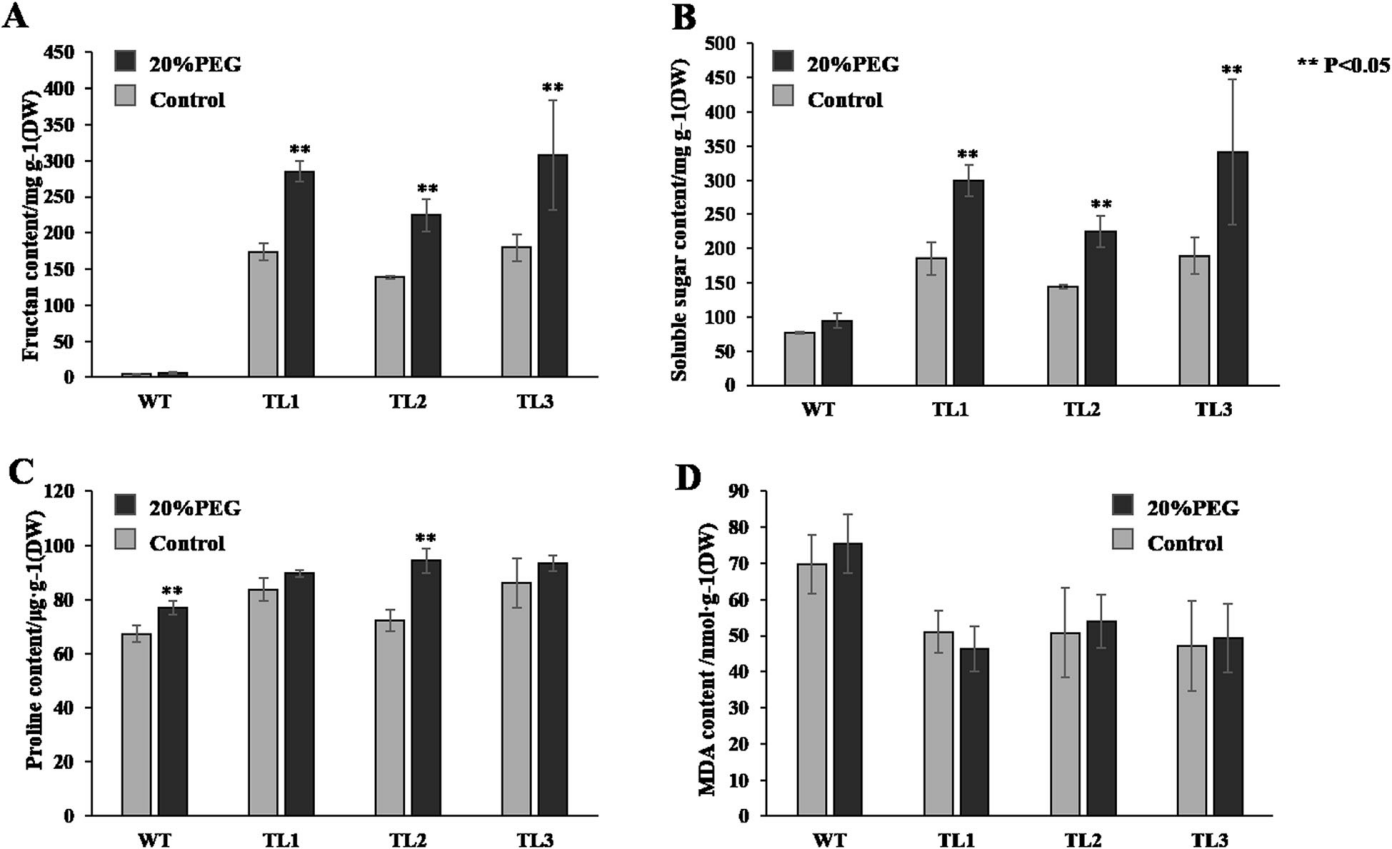
The physiological parameters (mean ± SE) of the transgenic and WT tobacco lines under the PEG-simulated drought stress (20% PEG) and control treatments. **a** Fructan concentration. **b** Soluble Suger concentration. **c** Proline concentration. **d** Malondialdehyde (MDA) concentration. WT: wild type; TL1 to TL3: transgenic lines; DW: dry weight; SE: standard error

To quantify PEG-simulated drought tolerance, the fructan, soluble sugar, proline, and MDA concentrations were measured in transgenic and WT tobacco lines under of stress (20% PEG) or control conditions (0% PEG). These parameters are closely associated with plant stress tolerance. In the control seedlings, the fructan concentration of the transgenic tobacco was in the range 173–180 mg·g^− 1^ dry weight (DW) (Fig. 3a), but little fructan was detected in the WT tobacco. The soluble sugar concentration in the transgenic lines ranged from 144.16 to189.60 mg·g^− 1^ (Fig. 3b), which was twice that in the WT plants. The proline concentrations in the transgenic lines were 72.24–86.18 μg·g^− 1^, slightly higher than that of the WT plants (Fig. 3c). The MDA concentrations were 47.18–51.03 nmol·g^− 1^ in the transgenic lines, which was significantly less than the corresponding values in the WT plants (Fig. 3d). After the plants were subjected to PEG-simulated drought stress of 20% PEG, the fructan concentration of the transgenic lines increased significantly, ranging from 224.48 to 307.62 mg·g^− 1^, compared with the control (173–180 mg·g^− 1^ DW), but no significant increase was observed in the WT plants after exposure to stress (Fig. 3a). Both soluble sugar and proline concentrations increased following treatment with 20% PEG, compared with the control. The soluble sugar concentrations were 224.48–341.13 mg·g^− 1^ in the transgenic lines and 94.71 mg·g^− 1^ in the WT (Fig. 3b). The proline content reached 89.60–94.29 μg·g^− 1^ and 77 μg·g^− 1^ in the transgenic and WT lines, respectively (Fig. 3c). There was no significant difference in the MDA concentration between the 20% PEG treatment and the control in either the transgenic or WT lines (Fig. 3d).

## Discussion

To understand the biological effects of the FTs from Jerusalem artichoke, the CDS of *Ht1-FFT* was isolated. It contained a 2025-bp open reading frame, and the predicted protein encoded 641 amino acids. The Ht1-FFT protein in the current study was very similar to the 1-FFT protein sequence of other plants from the Asteraceae, such as lettuce and chicory, and had some conserved structural features of the glycoside hydrolase family 32. Phylogenetic analysis showed that Ht1-FFT was clustered with the 1-FFTs of *L. sativa* and *C. intybus* among the dicots. The 1-FFTs and 1-SSTs belonged to different branches in the dicots. Moreover, the FTs in all dicots tested belonged to the same branch, whereas all the FTs in monocots were in the other branch. These results indicated that the *1-FFT* and *1-SST* genes became differentiated after the divergence of the monocots and dicots. More work needs to be carried out to better understand the evolution of the *FT* genes.

Overexpression of *FT*s has been used to increase the fructan concentration in several plant species. In the current study, overexpression of *Ht1-FFT* increased the fructan concentration in leaves of tobacco to 179.81 mg g^− 1^ DW under normal conditions, which was close to the upper limit of the fructan concentration in plant leaves. WT tobacco had a very low fructan concentration. Previous research had increased the fructan concentration in tobacco as a result of combinational transformation of three wheat genes encoding fructan biosynthesis enzymes [26]. The transgenic plants transformed with *Ta1-SST* + *Ta6-SFT* genes contained the highest fructan and soluble sugar concentrations, with both Ta1-SST and Ta6-SFT belonging to the S-type FTs, whereas transformation with the single F-type FT gene, *Ta1-FFT*, increased the fructan concentration only slightly. In the current study, transformation with the single F-type FT gene, *Ht1-FFT*, increased the fructan concentration dramatically. A possible explanation for this difference was that the FTs from the different species had different catalytic activities in tobacco.

Following the increase in fructan concentration under normal conditions, the concentrations of soluble sugars and proline also increased, whereas the MDA concentration decreased in the transgenic tobacco. PEG-simulated drought stress did not influence the fructan concentration of the WT tobacco, but increased the fructan concentration markedly in the transgenic lines. The WT appears to lack enough catalytic activity for fructan biosynthesis, which made it difficult to accumulate the fructan, even under PEG-simulated drought stress conditions. All transgenic lines showed greater PEG-simulated drought stress tolerance than WT plants under PEG-simulated drought stress, a finding which was similar to results from previous research [26]. When a fructan synthetase gene was transferred from a bacterium into tobacco, the fructan concentration of transgenic tobacco was significantly higher than that of the WT under PEG-simulated drought stress, with the PEG-simulated drought stress tolerance being significantly higher than that of the WT [27]. When the *1-SST* gene from lettuce was transferred into tobacco, the electrolyte leakage rate and malondialdehyde concentration of WT tobacco plants increased significantly under low temperature stress, indicating membrane damage, but the transgenic plants exhibited no significant change [20]. All the results obtained in the present study indicated that Ht1-FFT had high catalytic activity with respect to fructan biosynthesis, so that overexpression of *Ht1-FFT* could increase tolerance to PEG-simulated drought stress in tobacco.

## Conclusion

In this study, *Ht1-FFT* was important for the fructosidase domain-containing protein executing their catalysis activation. It was the close to *1-FFTs* from the *Lactuca sativa*, and *Cichorium intybus*. Expression levels of *Ht1-FFT* in transgenic tobacco were significantly higher than that of WT plants. The PEG-simulated drought resistance of transgenic plants was higher than WT. Fructan content of transgenic tobacco was higher than WT, but few fructan was detected in wild tobacco. *Ht1-FFT* gene from *Helianthus tuberosus* is an important gene implicated in fructan synthesis, and plays a positive role in PEG-simulated drought stress tolerance. Therefore, *Ht1-FFT* could be used in transgenic breeding for improvement of drought stress tolerance to improve drought resistance for crops.

## Methods

### Plant materials

The Jerusalem artichoke cultivar QY3 was used for *Ht1-FFT* isolation. Tobacco (*Nicotiana tabacum* L.) cv. Samsun was used as the host for transformation studies. Plants were grown in the greenhouses of the Northwest Plateau Institute of Biology, Chinese Academy of Sciences, at a temperature of 25 °C under a 12-h photoperiod.

### Isolation of the 1-FFT gene from Jerusalem artichoke

Total RNA was extracted from leaves using an RNAprep Pure Plant Plus Kit (Tiangen, China) and reverse transcribed to cDNA. Based on the sequence of *Ht1-FFT* obtained from our transcriptome analysis, primers were designed for amplifying *Ht1-FFT* gene (Tab S1). The PCR details were as follows: 2 min at 98 °C, followed by 35 cycles for 2 min at 98 °C, 30 s at 55 °C, and 2 min at 72 °C, with a final extension for 10 min at 72 °C. The PCR fragment was purified using the Universal DNA Purification Kit (Tiangen, China). The fragment was ligated to the pMD19-T vector (TaKaRa, China) and transformed into competent cells of *Escherichia coli* strain DH5α. The positive clones were sequenced by BGI (Shenzhen, China). All primers used in this research are listed in Tab S1.

### Bioinformatics analysis

The phylogenetic tree was constructed using the neighbor-joining method with MEGA4.0 [28]. The assembly and alignments were conducted using the Vector NTI 10 software package (Thermo Fisher Scientific, USA). Protein function annotations were carried out using the websites https://blast.ncbi.nlm.nih.gov/ and https://www.ebi.ac.uk/Tools/sss/ncbiblast/.

### Overexpression vector construction

According to the instructions in the Gateway cloning kit (Thermo Fisher Scientific, USA), the gene-specific primers FFT *att*B1 forward, FFT *att*B2 reverse, joint primers *att*B1 adapter and *att*B2 adapter were designed and synthesized. The complete *Ht1-FFT* gene coding sequence (CDS) was amplified from the pMD19-T vector ligated to the full-length *Ht1-FFT* CDS, using the gene-specific primers FFT *att*B1 forward and FFT *att*B2 reverse. Then, the amplified products were amplified by PCR, using the joint primers *att*B1 adapter and *att*B2 adapter. The vector pDONR207:Ht1-FFT was constructed by the BP reaction, and then the target gene *Ht1-FFT*, which had been recombined into pDONR207, was cloned into the expression vector pJAM1502 by the LR reaction, to obtain the overexpression vector pJAM1502:Ht1-FFT (Fig. S2).

### Generation of Ht1-FFT-overexpressing tobacco plants

The pJAM1502:Ht1-FFT plasmid was transferred into *Agrobacterium tumefaciens* strain LBA4404 by the freeze-thaw method [29]. The *Agrobacterium*-mediated leaf disk transformation method was performed to obtain transgenic tobacco [29]. Young tobacco leaves from plants at the 4- to 5-leaf stage were sterilized (75% alcohol for 1 min, followed by sodium hypochlorite for 12 min, then washed with sterile water three times), cut into 1 cm^2^ squares and placed into a suspension of *A. tumefaciens* for 10–15 min. The tobacco leaves were dried on aseptic filter paper and incubated on regeneration medium (Murashige & Skoog (MS) medium) for 2–3 days in the dark. Then, the dark-cultured tobacco leaves were transferred to induction medium for culture in the light. After 2 weeks of light culture, the leaves were transferred to subculture medium. When adventitious buds had grown to about 0.5 cm, they were transferred to rooting medium. After rooting was induced, the plantlets were transplanted to the greenhouse, and T0 tobacco lines with the *Ht1-FFT* gene were obtained by clonal propagation. For further experiments, the T3 family lines carrying objective gene without the separation were used.

### Molecular confirmation of transgenic tobacco

The genomic DNA (gDNA) was extracted from the putative tobacco transgenics, using the cetyltrimethylammonium bromide (CTAB) method. The gDNA of wild-type (WT) tobacco was used as the template, and target gene-specific primers (Tab S1) were used for PCR detection. The presence of the gene in transgenic tobacco was further verified by Reverse Transcription-PCR (RT-PCR), using gene-specific primers Rt FFT-F and Rt FFT-R, to amplify the *Ht1-FFT* gene, with Actin-F and Actin-R to amplify an actin fragment as an internal control. The RT-PCR amplification program was as follows: 94 °C for 2 min, 35 cycles at 94 °C for 30 s, at 55 °C for 30 s, and at 72 °C for 20 s, with a final extension at 72 °C for 10 min. The amplified products were detected by 1% agarose gel electrophoresis. One microgram of RNA was used to synthesize cDNA using the FastQuant RT kit (Tiangen, China) reverse transcriptase.

### PEG-simulated drought stress tolerance assay

The PEG-simulated drought stress experiment were carried out by exposing the plants at the 3- to 4-leaf stage to 10, 15% or 20% (w/v) polyethylene glycol (PEG) solution, whereas the control treatment (CK) was treated with pure water (0% PEG). Each treatment was replicated three times.

### Physiological parameter measurements

Carbohydrate concentration: a sample (0.25 g) was suspended in deionized water at 90 °C and incubated for 15 min. After cooling to room temperature, the extract was centrifuged at 3000×*g* for 5 min. The supernatant was retained and the pellet was re-suspended in deionized water and incubated again at 90 °C for 15 min before centrifugation. The two supernatants were combined. The supernatant sample was analyzed by high-performance liquid chromatography (HPLC) on a Shimadzu LC20A HPLC system, fitted with a LC10A differential refractive detector and a Shim-pack Sugar SCR-1011 column (Shimadzu, Japan). After the sample was filtered through a microporous filter membrane, it was injected onto the column at a volume of 5 μL. The mobile phase was ultrapure water. The flow rate was 1 ml min^− 1^ and column temperature was 80 °C. Quantification was performed using inulin from dahlia tuber (Fluka, Hungary) as an internal standard. The retention time was 5.76 min. The concentration of soluble sugars was the sum of fructan and reducing sugars.

Proline and malondialdehyde concentrations: proline and malondialdehyde (MDA) were quantified according to the manufacturer’s instructions of the matched test kit provided by the Nanjing Jiangcheng Bioengineering Institute (China).

The mean and standard error (SE) of the concentrations of fructan, soluble sugar, proline and MDA were calculated from three replicates. Significant differences were determined using analysis of variance (ANOVA) and Tukey’s honestly significant difference (HSD) test, where *P* < 0.05 was considered to be significant. All data were analyzed using SPSS software (IBM, USA).

## Supplementary information

**Additional file 1: Fig. S1.** The coding sequence and deduced protein of Ht1-FFT; **Fig. S2.** The diagram of the construct pJAM1502:Ht1-FFT; **Table S1.** The primers used in this research.

## Data Availability

All data generated or analysed during this study are included within the article and its additional files.

## Abbreviations

1-FFT: Fructan 1-fructosyl-transferase 1-FFT
MDA: Malondialdehyde
FTs: Fructosyltransferases
1-SST: 1- fructosyl transferase
6-SFT: Fructose-6-fructosyltransferase
6G-FFT: Fructose-6G-fructosyltransferase
DW: Dry weight
WT: Wild-type
RT-PCR: Reverse Transcription-PCR
CK: Control treatment
HPLC: High-performance liquid chromatography
SE: Standard error
ANOVA: Analysis of variance
HSD: Honestly significant difference
