# Supplementary material for: A fructan: the fructan 1-fructosyl-transferase gene from Helianthus tuberosus increased the PEG-simulated drought stress tolerance of tobacco
Source: Hereditas. 2020 Apr 20;157:14. doi: 10.1186/s41065-020-00131-3 (PMC7171796; doi:10.1186/s41065-020-00131-3)
Supplement: Supplementary file 1 — Additional file 1: Fig. S1. The coding sequence and deduced protein of Ht1-FFT; Fig. S2. The diagram of the construct pJAM1502:Ht1-FFT; Table S1. The primers used in this research. [file 41065_2020_131_MOESM1_ESM.docx]

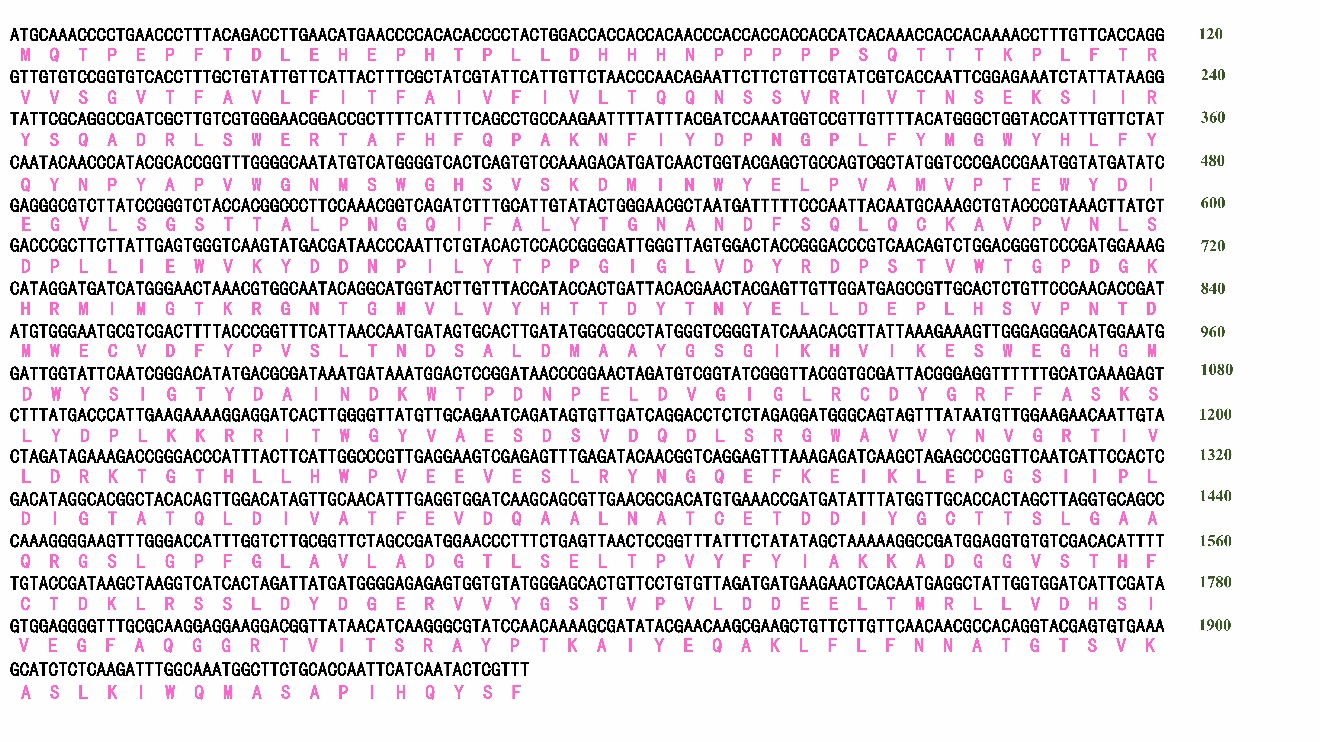


Figure S1. The coding sequence and deduced protein of Ht1-FFT.


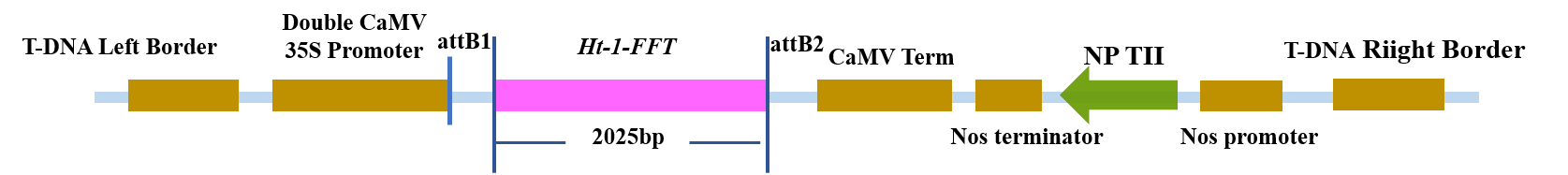
Figure S2. The diagram of the construct PJAM1502:Ht1-FFT

Table S1 The primers used in this research

| Name | Sequences |
| --- | --- |
| FFT attB1 forward | AAAAAGCAGGCTTCATGCAAACCCCTGAACCCTTTACAG |
| FFT attB2 reverse | AGAAAGCTGGGTCTTAAAAAGGGTATTGATGAA |
| attB1 adapter | GGGGACAAGTTTGTACAAAAAAGCAGGCT |
| attB2 adapter | GGGGACCACTTTGTACAAGAAAGCTGGGT |
| Rt FFT-F  Rt FFT-R  Actin-F  Actin-R | CGCTATCGTATTCATTGTTCTCAAC  ACAACTGACCATCTGGATCGTAA  AATGATCGGAATGGAAGCTG  TGGTACCACCACTGAGGACA |
